# Intermittent fasting alerts neurotransmitters and oxidant/antioxidant status in the brain of rats

**DOI:** 10.1007/s11011-024-01415-7

**Published:** 2024-09-18

**Authors:** Mona Abdel-Rahman, Aida A. Hussein, Omar A. Ahmed-Farid, Abdullah A. Sawi, Ahmed Esmat Abdel Moneim

**Affiliations:** 1 Zoology and Entomology Department, Faculty of Science, Helwan University, Cairo, Egypt; 2 Zoology and Entomology Department, Faculty of Science, Suez University, Suez, Egypt; 3 Department of Physiology, National Organization for Drug Control and Research (NODCAR), Giza, Giza Governorate Egypt

**Keywords:** Diet, Caloric restriction, Monoamines, Amino acids, BDNF

## Abstract

Several recent studies have attempted to understand how fasting has benefits for body health, especially the nervous system. To evaluate the impact of intermittent fasting on body weight, brain neurotransmitters, brain oxidative stress, and brain-derived neurotrophic factor (BDNF) in several areas of the brain, this study was conducted in rats. Thirty male Wistar rats were randomly divided into two groups. Group 1 (15 rats) served as the control and group 2 (15 rats) underwent intermittent fasting (IF; 24 h) for 1, 7, or 15 days. The findings demonstrated that intermittent fasting significantly reduced body weight. In this sense, brain monoamines and amino acids, namely dopamine, glutamate, aspartate, and oxidative stress markers (malondialdehyde and nitric oxide), decreased significantly after 1 day of IF. However, norepinephrine, serotonin, gamma-amino butyric acid, and glycine increased significantly. Additionally, glutathione levels were markedly elevated in IF. Surprisingly, the neuromodulatory effect of intermittent fasting fluctuates depending on the IF period. To support this fluctuation, BDNF levels increased after 1 day in the hippocampus and decreased after 15 days of intermittent fasting in all areas of the brain tested. In conclusion, our results show that intermittent fasting has beneficial influences on the brain; however, prolonged intermittent fasting can also induce some unfavorable physiological outcomes that prevent optimal neurological function.

**Supplementary Information:**

The online version contains supplementary material available at 10.1007/s11011-024-01415-7.

## Introduction

Fasting is a part of many religions and societies’ traditions. It is also frequently practiced and advised for rapid weight loss, as well as for the treatment and prevention of diseases (Solianik et al. 2016). The term intermittent fasting (IF) refers to a dietary pattern in which only the time allotted for eating is restricted, not the amount or type of food consumed. Although there are many other intermittent fasting regimes, the two most popular are alternate-day fasting (ADF) and time-restricted eating (TRE). TRE: Eating is limited to a certain period each day (for example, out of 24 h, 16 h are used for fasting and 8 h are used for eating).

ADF: Days of fasting and days of free eating alternate according to different plans; one of the most popular is the 5/2 method: The 5 − 2 fasting diet is a form of IF in which one fasts for two days and eats normally for five for calorie restriction. Modified alternate-day fasting is similar to ADF, but allows modest calorie intake (15–25% of caloric demand) on fasting days. Other types of fasting, such as those performed for religious or spiritual reasons, are also performed (Mattson et al. 2017; Patterson et al. 2015). Despite the fact that diet and calorie restriction are good for the brain (Gardener and Rainey-Smith 2018), such methods can be damaging to those who already have low body weight or muscle mass and are notoriously difficult for many people to maintain over time (Normandin et al. 2015).

Furthermore, numerous studies have shown that IF can increase the amount of neurogenesis in the hippocampus (Kim et al. 2018). Similarly, mice can live longer and have fewer age-related neurodegenerative disorders such as Alzheimer’s disease (AD) and ischemic stroke when they practice intermittent fasting (Mattson et al. 2017). According to previous research, fasting-induced metabolic alterations may improve brain function in terms of improved cognitive function, increased neuroplasticity, and resilience to damage and disease (Longo and Mattson 2014). During IF, several neuroprotective proteins, including protein chaperones such as heat shock protein 70 (HSP70), glucose-regulated protein 78 (GRP-78), neurotrophic factors such as brain-derived neurotrophic factor (BDNF) and fibroblast growth factor 2 (FGF2), and antioxidant enzymes such as superoxide dismutase and heme oxygenase-1, are up-regulated in animal models of stroke (Arumugam et al. 2010; Fann et al. 2014, 2017; Manzanero et al. 2014).

Therefore, the objective of the present study was to investigate how intermittent fasting significantly alters neurotransmitter levels, namely norepinephrine (NE), dopamine (DA), serotonin (5-HT), gamma-aminobutyric acid (GABA), glutamate (GLU), aspartate (ASP) and glycine (GLY), in the brain, the changes in neurotransmitter levels are dependent on the duration of the intermittent fasting regimen, and the effects of intermittent fasting on neurotransmitter levels vary across different brain regions. Furthermore, brain-derived neurotropic factor (BDNF) and oxidative stress biomarkers, namely malondialdehyde (MDA), nitric oxide (NO), and glutathione (GSH), were detected in different areas of the brain.

## Materials and methods

### Experimental animals

Thirty adult male Wistar rats (8–10 weeks old and weighing 150–200 g) were used in the current study. The Institutional Breeding House in Cairo, Egypt, provided the animals, which were then acclimated to standard laboratory conditions of a 12-hour light/dark cycle (lights on from 6:00 AM to 6:00 PM), 60% humidity, and 25 ° C. There was unlimited access to food and beverages. The National Institutes of Health Guide for the Care and Use of Laboratory Animals, 8th Edition (NIH publication no. 85 − 23, revised 1985), and all experimental protocols and procedures used in this study were approved by the Department of Zoology and Entomology, Faculty of Sciences, Helwan University (approval no. HU2021/Z/AAM0521-01).

### Study design

The rats were divided into two groups as follows:

- **Group I: Control Group (C):** Animals in this group (15 rats, 5 rats/cage) have been provided with food and water *ad libitum*.
- **Group II (Intermittent Fasting Group):** This set of animals (15 rats, 5 rats/cage) underwent an alternate-day fasting regimen in which they were given free access to food for 24 h and then disallowed for the next 24 h. This regimen was carried out for 15 days, starting at 9 a.m. and ending at 9 a.m. the following morning (Malinowski et al. 2019).

The animals were sacrificed on day 1 of the intermittent fasting period at 9 a.m., and then different brain areas (midbrain, thalamus and hypothalamus, and hippocampus) were quickly excised according to Glowinski and Iversen (1966) using ice plate weighted and stored at – 80 ° C for further investigations. The collection of samples was performed by blinding the experimenter.

Each piece of brain tissue was thoroughly mixed with 75% HPLC grade aqueous HPLC-grade methanol (10% w/v) (Arafa et al. 2016). The homogenate was spun at 4000 xg for 10 min and the supernatant was divided into two portions: the first was vacuum dried (70 Millipore) at room temperature for the determination of glutamate, aspartate, glycine and GABA (gamma-amino-butyric acid), while the second was utilized for the determination of monoamines.

### Determination of brain monoamine concentrations by the HPLC Method

The high performance liquid chromatography (HPLC) system included a 20-l loop UV variable wavelength detector, a quaternary pump, a column oven, and a rheodine injector. The data gathering program was acquired from Chemstation, and the report and chromatogram were taken from it. The solid phase extraction CHROMABOND column, NH2 phase, Catalog. No.: 730,031 (Macherey-Nagel, Dueren, Germany) was used to rapidly remove the sample from the lipids and trace elements. Subsequently, the sample was directly injected into a 150 × 2 mm AQUA 5 μm C18 125 column, Catalog. No.: 00 F-4331-B0 (bought from Phenomenex, Torrance, CA, USA) with the following operating parameters: mobile phase 20 mM potassium phosphate, pH 2.5, flow rate 1.5 ml/min, UV 210 nm. After 12 min, noradrenaline (NE), dopamine (DA), and serotonin (5-HT) were separated. The ensuing chromatogram revealed the position and concentration of each monoamine in the sample relative to the standard (DA (Catalog. No.: D-081), NE (Catalog No.: 1468501), and 5-HT (Catalog No. No.: H9523) were obtained from Sigma-Aldrich, St. Louis, MO, USA), as well as its quantity measured in µg per gram of brain weight (Pagel et al. 2000).

### Determination of brain amino acids

Brain glutamate, aspartate, glycine, and GABA were estimated by HPLC utilizing the precolumn PITC derivatization method according to the method of Heinrikson and Meredith (1984).

### Estimation of the BDNF protein

Brain-derived neurotrophic factor (BDNF) levels were measured using enzyme-linked immunosorbent assay kits according to the manufacturer’s instructions; Abcam, Cambridge, UK, Catalog. No.: ab213899).

### Evaluation of antioxidant activity

The thiobarbituric acid method described by Ohkawa et al. (1979) was utilized to measure malondialdehyde (MDA) lipid peroxidation. Griess reagent at 540 nm was used to measure the concentration of nitric oxide (NO) in various areas of the brain, as described by Green et al. (1982). Ellman’s reagent was used to compute the GSH levels and the result was a measurement of the yellow chromogen at 412 nm (Ellman 1959).

### Statistical analysis

The tests were carried out using SPSS version 23.0 (IBM Corp (2015), IBM SPSS Statistics for Windows, Version 23.0. IBM Corp, Armonk, NY, USA). One-way analysis of variance (ANOVA) was used to examine the relationships between the various groups, followed by Tukey’s post hoc analysis. Furthermore, to scrutinize the relationship between IF duration and diet regimen, 2-way ANOVA, followed by a least significant difference (LSD) test using SPSS software and graph paid prism, p values were reported. All parameters characterized by continuous data were subjected to Bartlett’s test to meet the homogeneity of the variance before analyzing the variance (ANOVA) and Dunnett’s t test. A Mann-Whitney U test was performed to calculate the significance. The data normality was first checked with the Shapiro–Wilk test. The results of homogeneity and normality were represented in the supplementary data (Tables S1-S4). In case of data nonnormality, the median and the upper and lower values were represented as supplementary data (Table S5). Standard deviation (SD) and the mean were used to present the data. At P-values of less than 0.05, the difference is deemed statistically significant. Histograms were produced using the Windows edition of GraphPad Prism (6.01, GraphPad Software, Boston, MA, USA).

## Results

### Changes in mean body weight after intermittent fasting

**Fig. 1:**
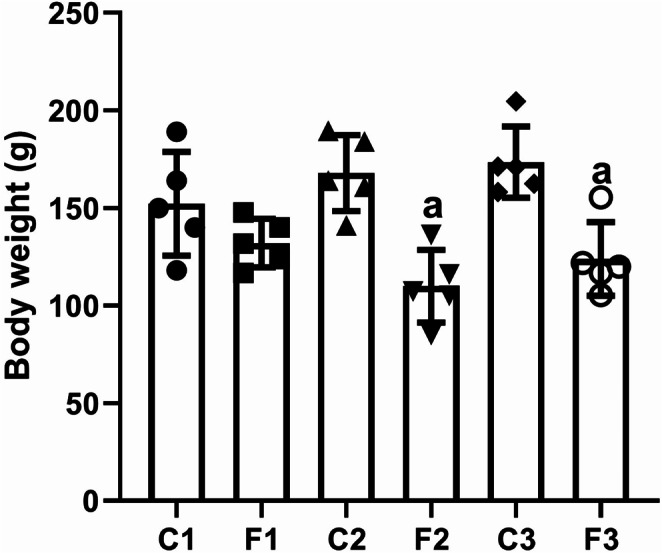
The mean body weight of rats exposed to IF (24 h alternate-day fasting) for one, seven, and fifteen days. Values are provided as means ± SD (*n* = 5). ^a^ reflects significant differences (*P* < 0.05) compared to control

The results obtained revealed a significant decrease in mean body weight (*F*(5, 24) = 8.494, *p* < 0.001, *W* = 0.418, *p* = 0.831) after one week (F2) and two weeks (F3) of IF compared to the control rats. Interestingly, the mean body weight of rats in the F3 group increased as evidenced by a nonsignificant elevation compared to the F2 group (*p* = 0.862) (Fig. 1). Furthermore, a two-way ANOVA revealed a nonsignificant change between the duration of IF and the diet regimen intervention (*F* = 2.601, *p* = 0.095).

### Monoamines alterations in different areas of the brain after intermittent fasting

**Fig. 2:**
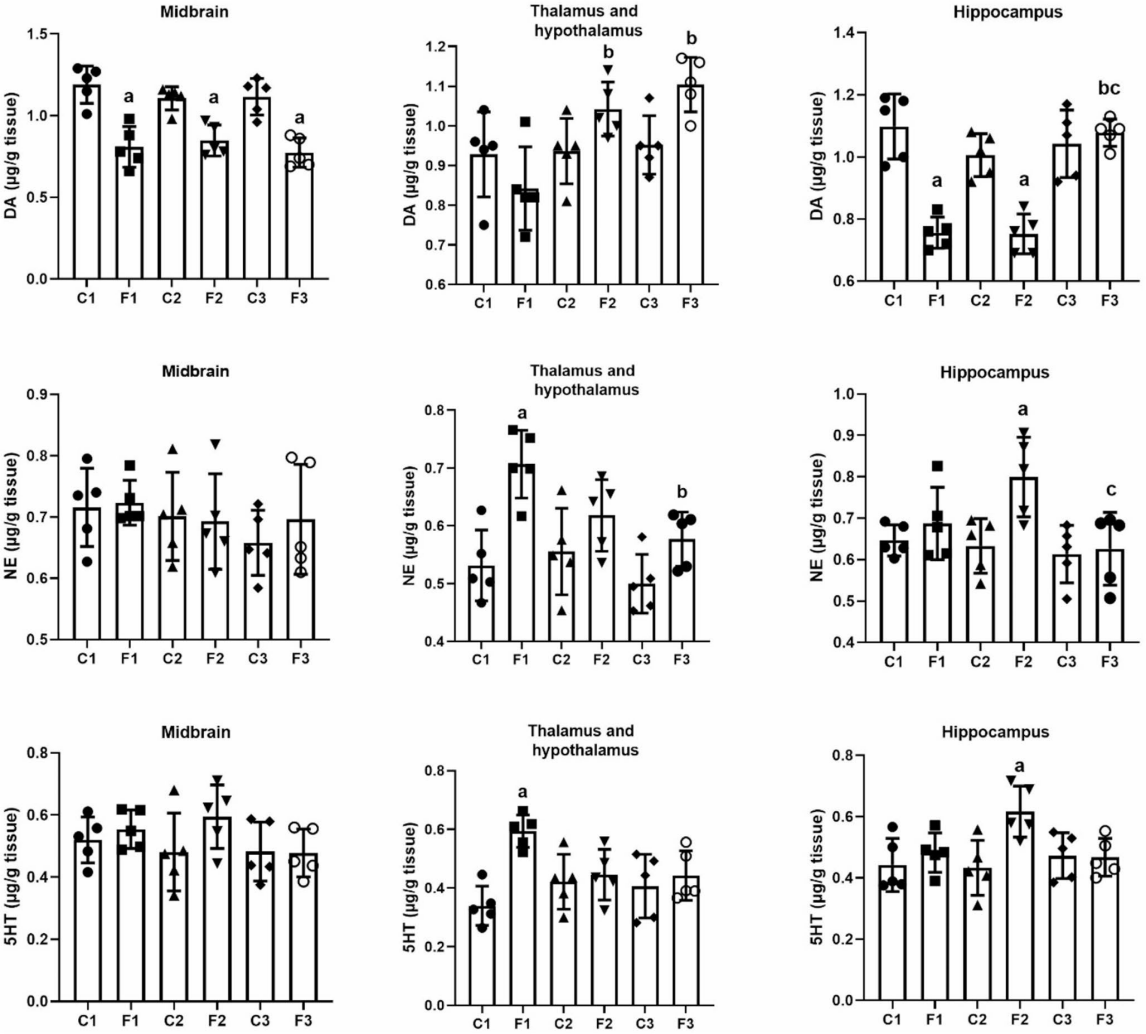
Dopamine, norepinephrine, and serotonin levels in different brain areas (midbrain, thalamus and hypothalamus, and hippocampus) in rats exposed to IF (24 h alternate-day fasting) for one, seven, and fifteen days. Values are provided as means ± SD (*n* = 5). ^a, b,^ and ^c^ reflect significant differences (*P* < 0.05) compared to the control, F1, and F2 groups, respectively

To elucidate the effect of IF on neurotransmitters, we determined the levels of DA, NE and 5-HT in different areas of the brain, namely, the midbrain, thalamus and hypothalamus, and hippocampus. The observed data showed a significant decrease in DA levels in the middle brain (*F*(5, 24) = 15.873, *p* < 0.001, *W* = 0.700, *p* = 0.629) and the hippocampus (*F*(5, 24) = 21.069, *p* < 0.001, *W* = 3.421, *p* = 0.018) at all time intervals except in the hippocampus of the F3 group. However, DA levels showed a nonsignificant change in the thalamus and hypothalamus (*F*(5, 24) = 5.821, *p* = 0.001, *W* = 0.146, *p* = 0.979) after 15 days of fasting intermittent (group F3) compared to control rats. Furthermore, the findings of the present study revealed a significant increase in DA levels in the F2 (*p* = 0.012) and F3 (*p* = 0.001) groups compared to the F1 group (one day of IF) in the thalamus and hypothalamus, and a significant increase in DA levels in the F3 group compared to the F1 (*p* < 0.001) and F2 (*p* < 0.001) groups in the hippocampus (Fig. 2). Furthermore, a two-way ANOVA analysis revealed a nonsignificant change in DA levels in the midbrain when comparing the duration of IF and the diet regimen intervention (*F* = 0.948, *p* = 0.402). On the other hand, a two-way ANOVA revealed a significant change in DA levels in the thalamus and hypothalamus, and hippocampus between the duration of IF and the diet regimen intervention (*F* = 5.428, *p* = 0.011) and (*F* = 16.282, *p* < 0.001), respectively.

Compared to the control group, NE levels showed a nonsignificant change in the midbrain (*F*(5, 24) = 0.564, *p* = 0.726, *W* = 1.135, *p* = 0.369) in all experimental groups. However, the data obtained showed a significant increase in NE levels in the thalamus and hypothalamus (*F*(5, 24) = 7.516, *p* < 0.001, *W* = 0.171, *p* = 0.971) after one day of IF (group F1), while there was a significant decrease in NE levels in the F3 (*p* = 0.023) group compared to the F1 group. Furthermore, there was a significant increase in NE levels in the F2 group in the hippocampus (*F*(5, 24) = 4.085, *p* = 0.008, *W* = 1.291, *p* = 0.301) compared to the control group, while NE levels decreased significantly in the F3 group compared to the F2 (*p* = 0.017) group (Fig. 2). Furthermore, the two-way ANOVA analysis revealed a nonsignificant change in NE levels in the midbrain, thalamus and hypothalamus, and hippocampus between the duration of IF and the diet regimen intervention (*F* = 0.306, *p* = 0.740), (*F* = 2.622, *p* = 0.093), and (*F* = 2.843, *p* = 0.078), respectively.

Figure 2 also shows a nonsignificant change in 5-HT level (*F*(5, 24) = 1.348, *p* = 0.279, *W* = 0.389, *p* = 0.852) in all time intervals compared to the control group in the midbrain. In the area of the thalamus and hypothalamus, the 5-HT level showed a significant increase (*F*(5, 24) = 4.961, *p* = 0.003, *W* = 0.914, *p* = 0.488) in the F1 group compared with the control group. Furthermore, the 5-HT level in the hippocampus increased significantly (*F*(5, 24) = 3.707, *p* = 0.013, *W* = 0.577, *p* = 0.717) in the F2 group compared to the control group. Furthermore, a two-way ANOVA analysis revealed a nonsignificant change in 5-HT levels in the midbrain between the duration of IF and the diet regimen intervention (*F* = 1.079, *p* = 0.356). On the other hand, a two-way ANOVA findings revealed a significant change in 5-HT levels in the thalamus and hypothalamus, and hippocampus between the duration of IF and the diet regimen intervention (*F* = 5.921, *p* = 0.008) and (*F* = 4.038, *p* = 0.03), respectively.

### Amino acids changes in different areas of the brain after intermittent fasting

**Fig. 3:**
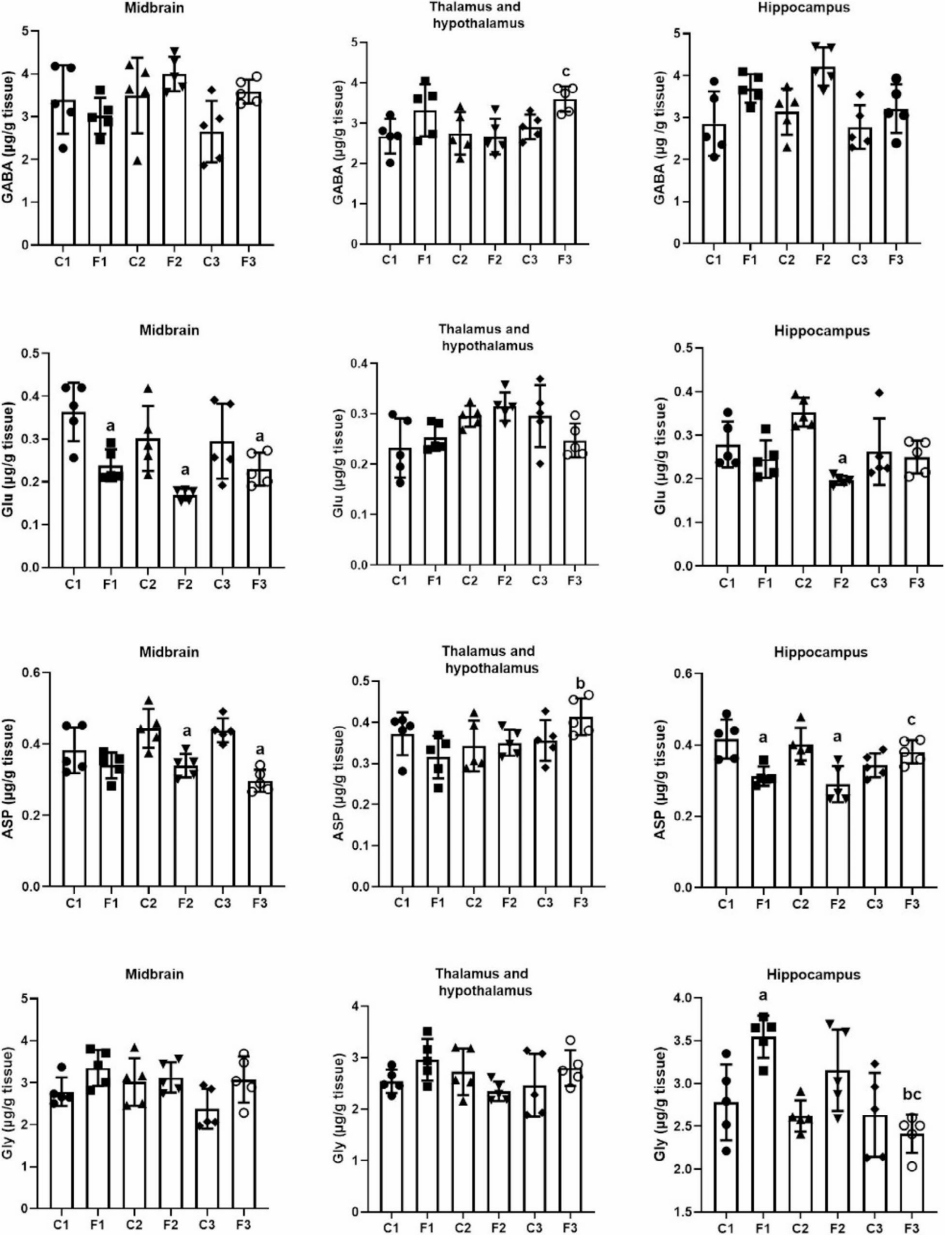
GABA, glutamate, aspartate, and glycine levels in different brain areas (midbrain, thalamus and hypothalamus, and hippocampus) in rats exposed to IF (24 h alternate-day fasting) for one, seven, and fifteen days. Values are provided as means ± SD (*n* = 5). ^a, b,^ and ^c^ reflect significant differences (*P* < 0.05) compared to the control, F1, and F2 groups, respectively

To elucidate the effect of IF on neurotransmitters, we determined the levels of GABA, GLU, ASP, and GLY in different areas of the brain, namely, the midbrain, thalamus and hypothalamus, and hippocampus. Compared to the control group, the present data showed a nonsignificant change in GABA levels in the midbrain (*F*(5, 24) = 2.793, *p* = 0.040, *W* = 1.252, *p* = 0.316) in all experimental groups. Furthermore, a nonsignificant change in GABA levels was detected (*F*(5, 24) = 3.545, *p* = 0.015, *W* = 1.577, *p* = 0.204) in all experimental groups in the thalamus and hypothalamus compared to the control group, however a significant increase in GABA levels in the F3 group compared to the F2 group (*p* = 0.039) group was detected. Furthermore, the findings of the present study revealed a nonsignificant change in GABA levels (*F*(5, 24) = 4.935, *p* = 0.003, *W* = 1.199, *p* = 0.339) in the F1, F2 and F3 groups compared to the control group in the hippocampus (Fig. 3). Furthermore, a two-way ANOVA results revealed a nonsignificant change in GABA levels in the midbrain, thalamus and hypothalamus, and hippocampus between the duration of IF and the diet regimen intervention (*F* = 2.873, *p* = 0.076), (*F* = 2.216, *p* = 0.131), and (*F* = 0.848, *p* = 0.441), respectively.

Figure 3 showed a significant decrease in the GLU level (*F*(5, 24) = 6.482, *p* = 0.001, *W* = 3.140, *p* = 0.025) in all experimental groups compared to the control group in the midbrain. In the thalamus and hypothalamus, the GLU level showed a nonsignificant change (*F*(5, 24) = 3.153, *p* = 0.025, *W* = 1.843, *p* = 0.142) at all time intervals compared to the control group.

Furthermore, the level of GLU in the hippocampus was significantly decreased (*F*(5, 24) = 6.056, *p* = 0.001, *W* = 2.129, *p* = 0.097) in the F2 group compared to the control group. Additionally, a two-way ANOVA findings revealed a nonsignificant change in GLU levels in the midbrain and, thalamus and hypothalamus between the duration of IF and the diet regimen intervention (*F* = 0.948, *p* = 0.401) and (*F* = 2.288, *p* = 0.123), respectively. However, a two-way ANOVA results revealed a significant change in GLU levels in the hippocampus between the duration of IF and the diet regimen intervention (*F* = 6.925, *p* = 0.004).

The results obtained (Fig. 3) revealed a significant decrease in ASP levels (*F*(5, 24) = 8.995, *p* < 0.001, *W* = 1.653, *p* = 0.184) in the F2 and F3 groups compared to the control group in the midbrain. However, ASP levels showed a nonsignificant change in the thalamus and hypothalamus (*F*(5, 24) = 2.217, *p* = 0.086, *W* = 0.736, *p* = 0.604) in all experimental groups, while there was a significant increase after 15 days of intermittent fasting (group F3) compared to group F2 (*p* = 0.05). Furthermore, the findings of the present study revealed a significant decrease in ASP levels (*F* 5, 24) = 7.241, *p* < 0.001, *W* = 1.282, *p* = 0.304) in the F1 and F2 groups compared to the control group in the hippocampus, but a significant increase in F3 compared to the F2 (*p* = 0.024) group. Furthermore, a two-way ANOVA analysis revealed a nonsignificant change in ASP levels in the midbrain and, thalamus and hypothalamus between the duration of IF and the diet regimen intervention (*F* = 3.258, *p* = 0.056) and (*F* = 3.376, *p* = 0.051), respectively. However, a two-way ANOVA results revealed a significant change in ASP levels in the hippocampus between the duration of IF and the diet regimen intervention (*F* = 10.074, *p* < 0.001).

Compared to the control group, the data obtained showed a nonsignificant change in the GLY level (*F*(5, 24) = 2.655, *p* = 0.048, *W* = 0.592, *p* = 0.706) in the midbrain after one, seven, and fifteen days of IF (F1,F2 and F3 groups). In the thalamus and hypothalamus, there was a nonsignificant change in the GLY level (*F*(5, 24) = 1.656, *p* = 0.184, *W* = 2.949, *p* = 0.033) in all groups compared to the control group. Furthermore, a significant increase in GLY levels was detected in the hippocampus (*F*(5, 24) = 6.525, *p* < 0.001, *W* = 2.548, *p* = 0.055) in the F1 group compared to the control group and a significant decrease in F3 compared to the F1 (*p* = 0.001) and F2 (*p* = 0.038) groups (Fig. 3). Furthermore, two-way ANOVA findings revealed a nonsignificant change in GLY levels in the midbrain and, thalamus and hypothalamus between the duration of IF and the diet regimen intervention (*F* = 1.137, *p* = 0.338) and (*F* = 3.046, *p* = 0.066), respectively. However, a two-way ANOVA revealed a significant change in GLY levels in the hippocampus between the duration of IF and the diet regimen intervention (*F* = 4.965, *p* = 0.016).

### Oxidative stress status in different areas of the brain after intermittent fasting

**Fig. 4:**
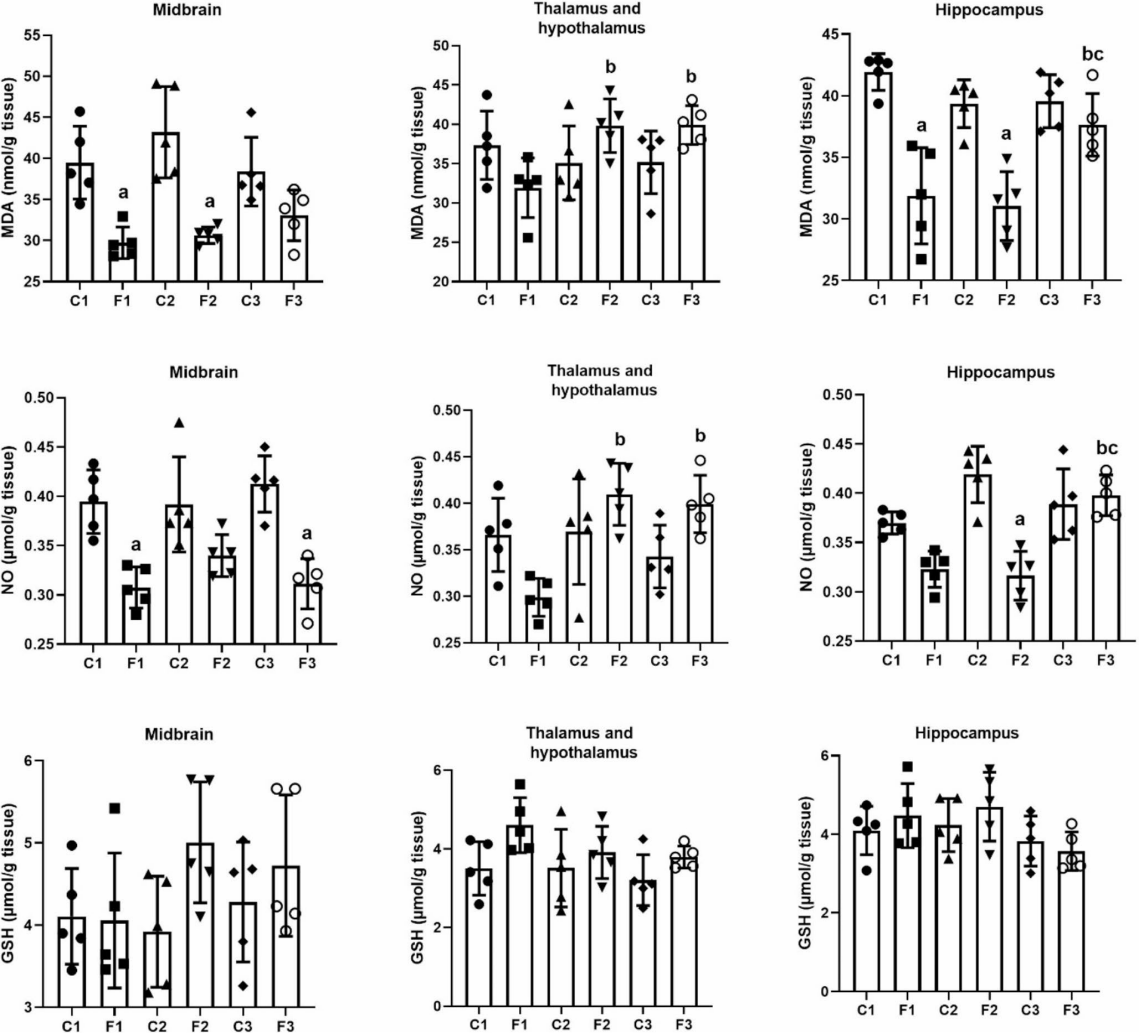
Oxidative stress biomarkers (MDA, NO, and GSH) levels in different brain areas (midbrain, thalamus and hypothalamus, and hippocampus) in rats exposed to IF (24 h alternate-day fasting) for one, seven, and fifteen days. Values are provided as means ± SD (*n* = 5). ^a, b,^ and ^c^ reflect significant differences (*P* < 0.05) compared to the control, F1, and F2 groups, respectively

To elucidate the effect of IF on biomarkers of oxidative stress, MDA, NO, and GSH levels were determined in different areas of the brain (Fig. 4). The results obtained revealed a significant decrease in MDA levels after one and seven days of IF and in NO levels after one and fifteen days in the midbrain (MDA: *F*(5, 24) = 10.644, *p* = 0.001, *W* = 3.186, *p* = 0.025; NO: (*F*(5, 24) = 10.986, *p* = 0.001, *W* = 0.702, *p* = 0.627) and a significant decrease in MDA levels after one and seven days of IF and a significant decrease in NO levels after 7 days of IF in hippocampus (MDA: *F*(5, 24) = 14.742, *p* < 0.001, *W* = 1.335, *p* = 0.283; NO: (*F*(5, 24) = 14.405, *p* < 0.001, *W* = 0.560, *p* = 0.730), while a significant increase was recorded in the F3 compared to the F1 groups (MDA: *p* = 0.018; NO: *p* = 0.001) and F2 (MDA: *p* = 0.005; NO: *p* = 0.001). In the thalamus and hypothalamus, MDA and NO levels showed a nonsignificant change (*F*(5, 24) = 3.233, *p* = 0.023, *W* = 0.342, *p* = 0.833; NO: (*F*(5, 24) = 5.766, *p* = 0.001, *W* = 0.496, *p* = 0.776) at all time intervals compared to the control group,. Furthermore, the F2 (MDA: *p* = 0.05; NO: *p* = 0.05) and F3 (MDA: *p* = 0.05; NO: *p* = 0.05) groups showed a significant increase in MDA and NO levels compared to the F1 group.

Furthermore, a two-way ANOVA analysis revealed a nonsignificant change in MDA levels in the midbrain between the duration of IF and the diet regimen intervention (*F* = 2.406, *p* = 0.112). On the other hand, a two-way ANOVA revealed a significant change in MDA levels in the thalamus and hypothalamus, and hippocampus between the duration of IF and the diet regimen intervention (*F* = 5.782, *p* = 0.009) and (*F* = 6.869, *p* = 0.004), respectively. Moreover, two-way ANOVA results revealed a nonsignificant change in NO levels in the midbrain between the duration of IF and the diet regimen intervention (*F* = 1.695, *p* = 0.205). On the other hand, a two-way ANOVA analysis revealed a significant change in NO levels in the thalamus and hypothalamus, and hippocampus between the duration of IF and the diet regimen intervention (*F* = 8.079, *p* = 0.002) and (*F* = 13.018, *p* < 0.001), respectively.

In comparison with the control group, the data obtained in Fig. 4 showed a nonsignificant change in GSH levels (*F*(5, 24) = 1.655, *p* = 0.184, *W* = 0.5000, *p* = 0.773) in the middlebrain and (*F*(5, 24) = 2.451, *p* = 0.063, *W* = 0.975, *p* = 0.453) in the thalamus and hypothalamus, and (*F*(5, 24) = 1.774, *p* = 0.156, *W* = 0.560, *p* = 0.730) in the hippocampus at all time intervals. Furthermore, a two-way ANOVA analysis revealed a nonsignificant change in GSH levels in the midbrain, thalamus and hypothalamus, and hippocampus between the duration of IF and the diet regimen intervention (*F* = 1.482, *p* = 0.247), (*F* = 0.691, *p* = 0.511), and (*F* = 0.807, *p* = 0.458), respectively.

### Modifications of the BDNF protein levels in different areas of the brain after intermittent fasting

**Fig. 5:**
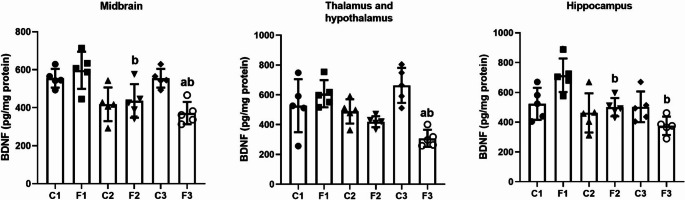
BDNF levels in different brain areas (midbrain, thalamus and hypothalamus, and hippocampus) in rats exposed to IF (24 h alternate-day fasting) for one, seven, and fifteen days. Values are provided as means ± SD (*n* = 5). ^a, b,^ and ^c^ reflect significant differences (*P* < 0.05) compared to the control, F1, and F2 groups, respectively

To elucidate the effect of IF on the BDNF protein, we determined the level of BDNF protein in different areas of the brain of albino rats. Compared to the control group, there was a significant decline in BDNF levels in the F3 group in all brain areas tested except the hippocampus (middle brain: *F*(5, 24) = 7.618, *p* < 0.001, *W* = 0.319, *p* = 0.897; thalamus and hypothalamus: *F*(5, 24) = 7.625, *p* < 0.001, *W* = 1.235, *p* = 0.316; hippocampus: *F*(5, 24) = 6.318, *p* < 0.001, *W* = 0.485, *p* = 0.784). Furthermore, compared to the F1 group, there was a significant decrease in BDNF levels in the F2 group in all brain areas tested except thalamus and hypothalamus (midbrain: (*p* = 0.02); thalamus and hypothalamus: (*p* = 0.05); hippocampus: (*p* = 0.025) (Fig. 5). Furthermore, a two-way ANOVA analysis revealed a significant change in BDNF levels in the midbrain, thalamus and hypothalamus, and hippocampus between the duration of IF and the diet regimen intervention (*F* = 6.923, *p* = 0.004), (*F* = 11.370, *p* < 0.001), and (*F* = 6.433, *p* = 0.006), respectively.

## Discussion

Although fasting diets and calorie restriction are beneficial for brain health, they are notoriously difficult for many people to maintain long-term and can be harmful to those who already have low body weight or muscle mass. However, a growing body of evidence from animal and human (observational and clinical research) investigations raises the intriguing possibility that fasting intervals without alterations to one’s calorie intake or diet may have comparable impacts on cognition and brain health (Gudden et al. 2021). Hence, in the present work, we examined the effect of IF on brain monoamines, amino acids, and BDNF levels in different brain regions of rats. The indicators of dopamine, glutamate, aspartate, and oxidative stress were shown to be dramatically reduced after 1 day of IF, according to the data obtained. However, there was a considerable increase in norepinephrine, serotonin, GABA, and glycine. Glutathione levels also increased significantly in IF. Unexpectedly, the neuromodulatory effect of IF varies with the IF period. BDNF levels increased after one day and reduced after 15 days of IF, supporting this fluctuation.

The IF diet causes observable metabolic alterations in the body (Wilhelmi de Toledo et al. 2019). After 12–36 h of fasting, the body enters a physiological condition called ketosis, which is characterized by low blood sugar levels, depleted liver glycogen stores, and the generation of ketone bodies by the liver generated from fat, which is a key source of energy for the brain and other tissues (Puchalska and Crawford 2017). The body also starts to use ketones that are produced as a result of fatty acid transformations (Camandola and Mattson 2017; Wilhelmi de Toledo et al. 2019). Whenever a person consumes fats, in the course of the lipolysis process, fatty acids are liberated from fat cells (Mattson et al. 2018) and then they are sent to the liver cells, where they undergo oxidation to create acetoacetate (AcAc) and beta-hydroxybutyrate (BHB), which are then released into the circulation and used as an energy source by cells (Camandola and Mattson 2017; Mattson et al. 2018).

Following the IF diet, the biochemical modifications of the lipids mentioned above lead to weight loss (Malinowski et al. 2019). Therefore, the decrease in body weight after 7 and 15 days of IF in the current study may be attributed to the lipolytic process and the reduction in meal frequency during fasting, which frequently causes a decrease in energy intake and a loss of body mass and body fat (Bernieh et al. 2010). On the other hand, Shawky et al. (2015) found that the total body weight did not change significantly during or after the fasting period. The present results clarified that the maximum decrease in mean body weight was found after 7 days, with a nonsignificant increase between the F3 and F2 groups. According to Anson et al. (2003), some mice that are fasted on alternate days have been shown to be able to eat twice as much on the feeding day, resulting in a net weekly calorie consumption that is comparable to mice fed *ad libitum*.

Reduced eating and body weight have a significant impact on the actions of dopamine circuits, which are important in many behaviors, including feeding. The activity of the mesolimbic dopamine system is altered by fasting and food restriction, which affects a variety of reward-related behaviors. Food restriction reduces baseline dopamine levels in several target areas and increases dopamine release in response to rewards like food and medications (Roseberry 2015). Dopamine neurons in the ventral tegmental area, VTA (a collection of neurons on the floor of the midbrain) release dopamine locally from their soma and dendrites in response to fasting, in addition to releasing it from their axons at efferent target sites (Beckstead et al. 2004; Lacey et al. 1987; Mercuri et al. 1997). These studies are consistent with the present results, which showed that IF caused a decrease in DA content in the midbrain and hippocampus, which may be due to an increase in its release in response to rewards.

To encourage a greater response to rewards, a prolonged increase in dopamine release in response to acute fasting can also increase the synchronization of VTA dopamine neurons (Carr 2007; Pan et al. 2006; Zhen et al. 2006). However, in the present study, there was an increase in DA content in the hypothalamus after 2 weeks of IF. According to Roseberry (2015), male and female rats exposed to the IF schedule had different levels of the catecholamine metabolite 3,4-dihydroxyphenylacetic acid in the cerebellum and an elevated DA content in the posterior region of the medial hypothalamus.

One of the key regulatory areas in this sense is the hypothalamus, which responds immediately to peripheral signals as well as input from the noradrenergic nuclei of the hindbrain (Itoi and Sugimoto 2010). The previous study by Gotthardt et al. (2016) demonstrated that the hypothalamic NE content increased as a consequence of the IF schedule. The interesting findings of the present study were the significant increase in NE level in the hypothalamus after 1 and 15 days and in the hippocampus after 7 days during IF. In acute glucoprivation settings, hyperphagia is frequently accompanied by elevated hypothalamic NE (Ritter et al. 2006). Adlouni et al. (1997) looked into how IF induces an increase in the hypothalamic norepinephrine content, which is followed by an increase in hyperphagia. The serotonergic system plays a role in behaviors that involve high cognitive demands. The cortex and hippocampus, two parts of the brain involved in learning and memory, both contain serotonin receptors (Meneses 1999).

One of the main signs of depression is low mood (Waraich et al. 2004). Antidepressants, primarily selective serotonin reuptake inhibitors or combination serotonin/norepinephrine reuptake inhibitors, are the main therapeutic options for treating depression (Cleare et al. 2015) leading to elevated synaptic levels of these monoamines (Morrissette and Stahl 2014). Many foods high in protein and dietary proteins contain the important amino acid tryptophan (Friedman and Levin 2012). The only precursor to serotonin produced both peripherally and centrally is tryptophan (Jenkins et al. 2016; Richard et al. 2009). Tryptophan hydroxylase converts L-tryptophan to 5-hydroxytryptophan once it has reached the central nervous system (CNS). 5-hydroxytryptophan is then decarboxylated to serotonin by the enzyme aromatic L-amino acid decarboxylase. The vesicular monoamine transporter then transports serotonin into vesicles (Jenkins et al. 2016).

In the present study, the increase in the 5-HT content in the hypothalamus after 1 day and the hippocampus after 1 week of the IF schedule may be due to the increased availability of tryptophan during fasting, which increased serotonin synthesis. According to Martin et al. (2007), compared to rats fed ad libitum, female rats exposed to 40% caloric restriction performed better on a behavioral cognitive task and had higher serotonin and lower DA levels in the hippocampus. Additionally, mice exposed to ketones for five days have been shown to display enhanced spatial memory and learning. These results are in parallel with the present results, where IF caused a decrease in DA and an increase in 5-HT content in the hippocampus after 7 days.

In the study conducted by Erecinska et al. (1996), the accumulation of GABA in the synaptic terminals of rats on a ketogenic diet was examined. An earlier study demonstrated that caloric restriction increases hypothalamic GABAergic neurons, whereas fasting decreases GABA release from neurons (Jarvie et al. 2017). These are in agreement with the present results, where the GABA content increased in the tested areas at different times in the IF schedule. The ketogenic diet reduced glutamate levels and increased GABA, which reduced excitability and seizures in the hippocampus (Olson et al. 2018). This is in agreement with the present results, where glutamate content decreased in the midbrain and hippocampus. The most effective time was 7 days of IF.

According to Yudkoff et al. (2007), because the ketone body must be converted to acetyl-CoA, which is then processed by citrate synthase to produce citrate and CoA (acetyl-CoA + oxaloacetate, citrate + CoA), there is less oxaloacetate available for the transamination of glutamate to aspartate. The glutamate decarboxylase pathway can then use glutamate to produce GABA in GABA-ergic neurons. Additionally, blood acetate levels increase during ketosis. Acetate is consumed primarily by astrocytes, where it is converted to glutamine, which can be transported to GABA-ergic neurons and converted to GABA. The previous studies and the present results may explain the decrease in glutamate content, which may be due to its conversion into GABA by glutamate decarboxylase, which led to increased GABA content. In addition, decreasing the release of GABA and converting acetate to glutamine led to an increase in GABA content. Furthermore, the limited availability of oxaloacetate for the transamination of glutamate to aspartate may be the cause of the decrease in Asp content in the midbrain and hippocampus.

The brainstem, spinal cord, hypothalamus, hippocampus, and retina of animals from various phyla exhibit the highest levels of glycine, an essential and extensively distributed inhibitory neurotransmitter (de Bartolomeis et al. 2020; Schousboe and Waagepetersen 2006). Glycine is synthesized primarily from serine and threonine (Ducker and Rabinowitz 2017). In the CNS, serine hydroxymethyltransferase catalyzes the synthesis of glycine from serine (de Bartolomeis et al. 2020). Felig et al. (1969) found that after the first five days of fasting, plasma levels of glycine, threonine, and serine gradually increased. This may have increased glycine synthesis, as seen in the present data in the midbrain and hippocampus from the first day of IF.

Free radicals are extremely reactive substances because they have unpaired valence electrons (Lobo et al. 2010). Reactive nitrogen species (RNS) and reactive oxygen species (ROS) include dangerous free radicals such as nitric oxide and superoxide, respectively (Ozkul et al. 2007). When antioxidative processes are out of balance, these reactive molecules can damage DNA, proteins, and lipids, resulting in cell toxicity and tissue damage (Salim 2017). The selectivity of the blood-brain barrier, which limits the passage of some antioxidants such as vitamin E, and the excessive consumption of polyunsaturated fatty acids are two factors that make the central nervous system particularly susceptible to the action of ROS (Shukla et al. 2011). Neurodegenerative processes are triggered by high ROS levels leading to oxidative damage (Jelinek et al. 2021).

According to Bales and Kraus (2013) and Walsh et al. (2014), calorie restriction, including fasting, is an effective way to reduce the impact of oxidative stress. There are other fasting plans; however, IF is one of the plans that has received the most research (Sanvictores et al. 2023). The positive benefits of IF are supported by improvements in mitochondrial function and decreases in cellular oxidative stress (Amigo and Kowaltowski 2014; Raefsky and Mattson 2017). Increasing the turnover rate of oxidized macromolecules or antioxidant enzyme activity, as well as lowering LDL cholesterol (De Cabo et al. 2004). A recent study by Hardiany et al. (2022) concluded that oxidative stress is decreased by fasting. Furthermore, the study of Singh et al. (2015) showed that compared to middle-aged rats, IF improved motor coordination and reduced oxidative damage by reducing protein carbonylation in the cortex, hippocampus, and hypothalamus. However, de Souza et al. (2024) found that IF for 15 days can increase oxidative stress to decrease memory. But others found that combining physical exercise with IF can block the negative effects of IF on memory and anxiety (Braz et al. 2023).

In the present study, IF caused a reduction in lipid peroxidation (MDA) and NO levels in the midbrain after all times tested, including the hypothalamus after 1 day, and the hippocampus after 1 and 7 days. Also, IF caused an increase in glutathione in the midbrain after 7 days and in the hypothalamus after 1 and 15 days. Malondialdehyde is an end product created during this lipid peroxidation as a result of the breakdown of phospholipids in the cell membrane. MDA is a powerful indicator of lipid oxidation that is released into the extracellular space before entering the blood (Dalle-Donne et al. 2006; Draper and Hadley 1990). Hazzaa et al. (2020) reported that IF significantly increased GSH concentrations in the hippocampus, decreased MDA concentrations, and improved memory function.

Calabro et al. (2011) have shown that high dosages of L-arginine are necessary to ensure proper NO synthesis in the brain. However, fasting simultaneously decreased the level of arginine in the blood (Charkey et al. 1955). These findings may explain the reduction in NO content in the present results. However, the present results showed a significant increase in hypothalamic NO after 15 days, and at the same time, there were significant increases between F3 (15 days) and F1 (1 day) in MDA and NO content in the hypothalamus and hippocampus. Additionally, there is a significant decrease in GSH after 15 days in the hypothalamus. At the same time, there are significant decreases between F3 and F1 in the hypothalamus and hippocampus in GSH content, which may be due to the longer fasting time.

The previous study of Hardiany et al. (2022) demonstrated that GSH content was greater in IF (18/6) than in PF (40/8) for 6 days. According to Stankovic et al. (2013), MDA concentrations were only started to increase at the end of the 7-day fasting regimen and were much higher in the livers of Wistar rats given less than 50% of the recommended daily caloric intake than in those given 60% or more. To maintain homeostasis, the body must adapt to stressful circumstances such as fasting and other forms of calorie restriction (Hardiany et al. 2022). According to Ristow and Zarse (2010), the stress brought on by calorie restriction can cause the cellular defense system known as hormesis to activate. To avoid the cell from failing to respond to a limited energy supply, this stressor must be present in moderation. If not, oxidative stress increases. So, the increase in MDA and NO and the decrease in GSH in the present results after 15 days may be due to the cell not adapting and oxidative stress rises as an indicator of maladaptation to the long fasting period.

The formation of new neurons in the brain is referred to as neurogenesis. Under the influence of many neurotrophic stimuli, the hippocampus is typically the main site of neurogenesis. The most common neurotrophic factor is BDNF, often known as the growth hormone of the brain. Exercise, sleep, aging, and diet practices are some elements that have an impact on BDNF expression (Akagi et al. 2015; Garcia et al. 2010; Sakr et al. 2015; Weissmiller and Wu 2012). Increases in neurotrophic factors and anti-inflammatory cytokines are also caused by fasting cycles, which decrease hippocampal cell death and promote neurogenesis (Arumugam et al. 2010; Marosi and Mattson 2014). According to a previous study, increasing these components by calorie restriction or fasting may improve brain function (Angelova et al. 2013). Fasting and ketogenic diets may protect neurons of the hippocampus from seizure-related damage (Greco et al. 2016; McNally and Hartman 2012; Sampaio 2016).

BHB is believed to be the predominant energy source for neurons when blood glucose levels are low, such as while fasting for more than 12 h in mice or 24 h in humans [glycogen provides glucose for roughly 24 h in humans] (Chowdhury et al. 2014; Green and Bishop 2019). BHB induces the expression of the BDNF gene and increases the levels of BDNF protein in neuronal cells of the cerebral cortex neuronal cells (Marosi et al. 2016). Ketone bodies play a crucial signaling role in hippocampal and cortical neurons by promoting BDNF transcription through the regulation of histone deacetylase, an enzyme that inhibits the expression of BDNF (Elesawy et al. 2021).

The present results indicated that IF caused an increase in BDNF levels in the hippocampus after one day; this result is in agreement with the study of Duan et al. (2001) which examined how rats on a dietary restriction (DR) program had significantly increased amounts of BDNF in their hippocampus, cerebral cortex, and striatum compared to controls. They also added that intermittent fasting and exercise induce neurogenesis through increasing BDNF (Marosi and Mattson 2014). The increase in BDNF could be related to the reduction of histone deacetylase activity by BHB, which increases BDNF expression. On the other hand, the present results show that IF caused a reduction in BDNF in all regions tested after 15 days, which may be due to glucose depression after a long fasting period.

It is crucial to recognise that other studies have already looked into how intermittent fasting affects neurotrophic factors and neurotransmitters. For example, Mattson et al. (2020) examined how IF affected cognitive function and BDNF levels. Li et al. (2013) investigated how IF affected the expression of dopamine receptors. Furthermore, research like that done by Harvie and Howell (2017) has evaluated the functional impact of IF by combining behavioural measurements and biochemical investigations. Our results on amino acid and neurotransmitter alterations support and add to these earlier findings. Our findings support previous research, but they also offer a more thorough examination of various neurotransmitters and brain areas throughout varying IF durations, which adds to our understanding of the complex neurochemical effects of IF. Indeed, the physiological benefits of lifestyle interventions improve the systemic metabolic profile and strongly correlate with improved cognitive function and improved nerve function (O’Brien et al. 2017).

### Study limitations

It should be highlighted that the lack of an a priori sample size calculation was one of the study’s limitations. The sample size was established based on comparable past research and established procedures in our laboratory. This limitation can impact the study’s statistical power and the validity of the findings. This restriction should be addressed in future studies by formal power analyses to establish suitable sample sizes.

## Conclusions

From current findings and previous research, it is possible to conclude that intermittent fasting may lead to a reduction in body weight, which may be due to an increased lipolytic process. IF increases some brain neurotransmitters (norepinephrine, serotonin, GABA, and glycine), which may be due to increased synthesis or decreased release of these. At the same time, it reduces dopamine, glutamate, and aspartate, which may be due to decreased synthesis or increased release. IF caused an increase in BDNF levels in the hippocampus after one day, which may lead to increased neurogenesis. It also increased GSH and decreased MDA and NO after seven days of intermittent fasting, which may be due to a decrease in oxidative damage. Therefore, IF may be beneficial in improving mood, memory function, and motor coordination. However, IF caused an increase in NO and a decrease in BDNF levels after 15 days, which may be due to the harmful effect of intermittent fasting for an extended period of time.

## Supplementary Information

Below is the link to the electronic supplementary material.

Supplementary file1 (PDF 253 kb)

## Data Availability

All data generated or analyzed during this study are included in this published article.
