# Supplementary material for: Intermittent fasting alerts neurotransmitters and oxidant/antioxidant status in the brain of rats
Source: Metab Brain Dis. 2024 Sep 18;39(7):1291–305. doi: 10.1007/s11011-024-01415-7 (PMC11513736; doi:10.1007/s11011-024-01415-7)
Supplement: Supplementary file 1 — Supplementary file1 (PDF 253 kb) [file 11011_2024_1415_MOESM1_ESM.pdf]

Supplementary data

Table S1: Results of homogeneity and normality of the body weight.

Levene Statistic	df1	df2	Sig.
0.418	5	24	0.831

Table S2: Results of homogeneity and normality of neurotransmitters, oxidative stress, and BDNF in midbrain.

Parameter	Levene Statistic	df1	df2	Sig.
DA	0.70	5	24	0.629
**NE**	1.135	5	24	0.369
**5-HT**	0.389	5	24	0.852
**GABA**	1.252	5	24	0.316
**GLU**	3.140	5	24	0.025
**ASP**	1.653	5	24	0.184
**GLY**	0.592	5	24	0.706
**MDA**	3.168	5	24	0.025
**NO**	0.702	5	24	0.627
**GSH**	0.50	5	24	0.773
**BDNF**	0.319	5	24	0.897

Table S3: Results of homogeneity and normality of neurotransmitters, oxidative stress, and BDNF in thalamus and hypothalamus.

Parameter	Levene Statistic	df1	df2	Sig.
**DA**	0.146	5	24	0.979
**NE**	0.171	5	24	0.971
**5-HT**	0.914	5	24	0.488
**GABA**	1.577	5	24	0.204
**GLU**	1.843	5	24	0.142
**ASP**	0.736	5	24	0.604
**GLY**	2.949	5	24	0.033
**MDA**	0.342	5	24	0.883
**NO**	0.496	5	24	0.776
**GSH**	0.975	5	24	0.453
**BDNF**	1.253	5	24	0.316

Table S4: Results of homogeneity and normality of neurotransmitters, oxidative stress, and BDNF in hippocampus.

Parameter	Levene Statistic	df1	df2	Sig.
**DA**	3.421	5	24	0.018
**NE**	1.291	5	24	0.301
**5-HT**	0.577	5	24	0.717
**GABA**	1.199	5	24	0.339
**GLU**	2.129	5	24	0.097

**ASP**	1.282	5	24	0.304
**GLY**	2.548	5	24	0.055
**MDA**	1.335	5	24	0.283
**NO**	0.908	5	24	0.492
**GSH**	0.560	5	24	0.730
**BDNF**	0.485	5	24	0.784

Table S5: Dopamine levels in midbrain in rats exposed to IF (24 hours alternate-day fasting) for one, seven, and fifteen days.

**Group**	**Median**	**Upper value**	**Lower value**
**C1**	1.23	1.01	1.29
**F1**	0.78	0.66	0.98
**C2**	1.13	0.98	1.16
**F2**	0.8	0.76	0.97
**C3**	1.17	0.96	1.23
**F3**	0.74	0.69	0.88
